# Exhaled phospholipid transfer protein and hepatocyte growth factor receptor in lung adenocarcinoma

**DOI:** 10.1186/s12931-022-02302-4

**Published:** 2022-12-21

**Authors:** Jesper Andreasson, Embla Bodén, Mohammed Fakhro, Camilla von Wachter, Franziska Olm, Malin Malmsjö, Oskar Hallgren, Sandra Lindstedt

**Affiliations:** 1 Department of Cardiothoracic Surgery, Skåne University Hospital, Lund, Sweden; 2 Department of Clinical Sciences, Lund University, Entrégatan 7, 22242 Lund, Sweden; 3 Department of Cardiothoracic Surgery, Rigshospitalet, University of Copenhagen, Copenhagen, Denmark; 4 Ludwig-Maximilians-University, Munich, Germany

**Keywords:** Exhaled breath particles, Hepatocyte growth factor receptor, Lung cancer, Particle flow rate, Phospholipid transfer protein, Lung adenocarcinoma

## Abstract

**Background:**

Screening decreases mortality among lung cancer patients but is not widely implemented, thus there is an unmet need for an easily accessible non-invasive method to enable early diagnosis. Particles in exhaled air offer a promising such diagnostic tool. We investigated the validity of a particles in exhaled air device (PExA) to measure the particle flow rate (PFR) and collect exhaled breath particles (EBP) to diagnose primary lung adenocarcinoma (LUAD).

**Methods:**

Seventeen patients listed for resection of LUAD stages IA–IIIA and 18 non-cancer surgical control patients were enrolled. EBP were collected before and after surgery for LUAD, and once for controls. Proteomic analysis was carried out using a proximity extension assay technology. Results were validated in both plasma from the same cohort and with microarray data from healthy lung tissue and LUAD tissue in the GSE10072 dataset.

**Results:**

Of the 92 proteins analyzed, levels of five proteins in EBP were significantly higher in the LUAD patients compared to controls. Levels of phospholipid transfer protein (PLTP) and hepatocyte growth factor receptor (MET) decreased in LUAD patients after surgery compared to control patients. PFR was significantly higher in the LUAD cohort at all timepoints compared to the control group. MET in plasma correlated significantly with MET in EBP.

**Conclusion:**

Collection of EBP and measuring of PFR has never been performed in patients with LUAD. In the present study PFR alone could distinguish between LUAD and patients without LUAD. PLTP and MET were identified as potential biomarkers to evaluate successful tumor excision.

**Supplementary Information:**

The online version contains supplementary material available at 10.1186/s12931-022-02302-4.

## Background

Lung cancer is the leading cause of cancer mortality, causing almost 1.8 million deaths worldwide in 2020 and accounting for 18% of all cancer deaths [1]. Despite advances in treatment, 5-year survival is poor, ranging from above 25% for women with non-small cell lung cancer (NSCLC) to below 5% for men with small-cell lung cancer in the Swedish National Lung Cancer Registry [2]. Most lung cancers are detected in advanced stages when curative treatment is no longer an option. Screening with low-dose computed tomography has proven effective in two large, randomized trials, with successful early detection and reduced mortality [3, 4]. However, lung cancer screening is not widely implemented due to difficult logistics, overdiagnosis and false-positive findings [5, 6]. The addition of risk-based screening models based on sociodemographic factors, clinical symptoms and biomarkers, alone or combined, could enhance the efficacy of lung cancer screening. Biomarkers can be found in different sample types, such as blood, urine, or exhaled air, and can be based on circulating cells, nucleic acids, proteins, or other molecules. Despite great efforts to identify suitable biomarkers, no method is yet established in clinical use due to lack of significant improvement in predictive performance, and sampling procedures can be complex and costly [7]. However, there are some proteins of interest that have a known connection to LUAD. One of these is hepatocyte growth factor receptor (MET). In approximately 70% of LUAD tumor tissue the MET gene is significantly overexpressed [8], moreover, overexpression of MET in plasma of patients with LUAD is a known phenomenon [9]. Given this, we investigated the correlation between levels of MET in EBP and s-MET in plasma in this study.

Collection of exhaled air allows for investigation of exhaled breath particles (EBP) [10], volatile organic compounds [11] and exhaled breath condensate (EBC) [12, 13], offering a unique isolated matrix of the respiratory system for biomarker analysis. Volatile organic compounds might reflect the presence of neoplasms or disease processes not specific to the lungs, and EBC is hampered by salivary contamination, whereas EBP reflect the distal airways selectively [14, 15], which is why EBP have gained much interest as a potential source of biomarkers. The respiratory tract lining fluid (RTLF) covers the epithelial surfaces of the distal airways. As small airways and alveoli open and close, particles from the RTLF enter the large airways and are subsequently exhaled [16]. The particle flow from the airways is measured as the particle flow rate (PFR) of EBP, which are collected for subsequent analysis [14, 15, 17]. In this study, patients undergoing lung cancer surgery for lung adenocarcinoma (LUAD) and patients without LUAD were enrolled. We aimed to evaluate proteins in EBP as well as analyzing PFR before and 1 month after surgery and comparing the results between LUAD patients and control patients, thereby evaluating the potential to use EBP and PFR as diagnostic tools. Analyzing EBP and PFR have previously been proven useful in diagnosing and evaluating other pulmonary diseases such as asthma, acute respiratory distress syndrome (ARDS) and primary graft dysfunction (PGD) in lung transplant recipients and in patients with COVID-19 by us and other research groups [10, 14, 15, 17–19].

## Patients and methods

This study is a prospective observational clinical trial with the aim to analyze exhaled breath particles from patients with LUAD and control patients to identify protein biomarkers and evaluate their potential usefulness in diagnosing and evaluating surgical resection of LUAD. We also aimed to investigate differences in particle flow rate between patients with LUAD and control patients. The study is approved by the Swedish Ethical Review Authority (Dnr. 2017/519). All patients signed a written, informed consent prior to enrollment.

### Patient demographics

A total of 35 patients were included: 17 LUAD patients scheduled for resection and 18 patients without LUAD scheduled for other non-cancer surgery, referred to as the control group. Inclusion criteria were TNM staging system up to pTNM N2/IIIA (TNM 7th edition [20]). Follow-up with survival was recorded 3 years after surgery. Demographic data are shown in Table 1 and the histopathological stage of the LUAD patients is shown in Table 2. A flow chart of enrolled subjects is shown in Additional file 1: Fig. S1.

**Table 1:** Patient characteristics

	All patients (n = 35)	LUAD (n = 17)	Control (n = 18)	Significance
Sex				
Male	21 (60)	6 (35)	15 (83)	p = 0.0059
Female	14 (40)	11 (65)	3 (17)	p = 0.0059
Age, years	70 (43–83)	72 (59–83)	68 (43–80)	p = 0.3381
BMI, kg/m^2^	27.5 (19.6–38.0)	27.4 (21.1–35.6)	27.6 (19.6–38.0)	p = 0.8799
Comorbidities				
Coronary artery disease	14 (40)	2 (12)	12 (67)	p = 0.0016
Diabetes mellitus	13 (37)	3 (18)	10 (56)	p = 0.0354
Hypertension	22 (63)	8 (47)	14 (78)	p = 0.0858
WHO performance status prior to surgery				
0	13 (37)	9 (53)	4 (22)	p = 0.0858
1	15 (43)	8 (47)	7 (39)	p = 0.7380
2	7 (20)	0 (0)	7 (39)	p = 0.0076
Smoking history				
Current	2 (6)	2 (12)	0 (0)	p = 0.2286
Former (> 6 weeks)	26 (74)	11 (65)	15 (83)	p = 0.2642
Never	7 (20)	4 (23)	3 (17)	p = 0.6906

Characteristics for patients with lung adenocarcinoma (LUAD) and non-cancer surgical control patients. All data are reported as n (%) or mean (range). *BMI* body mass index. Significance was defined as: p < 0.0001 (****), p < 0.001 (***), p < 0.01 (**), p < 0.05 (*), and p > 0.05 (not significant, ns)

**Fig. 1:**
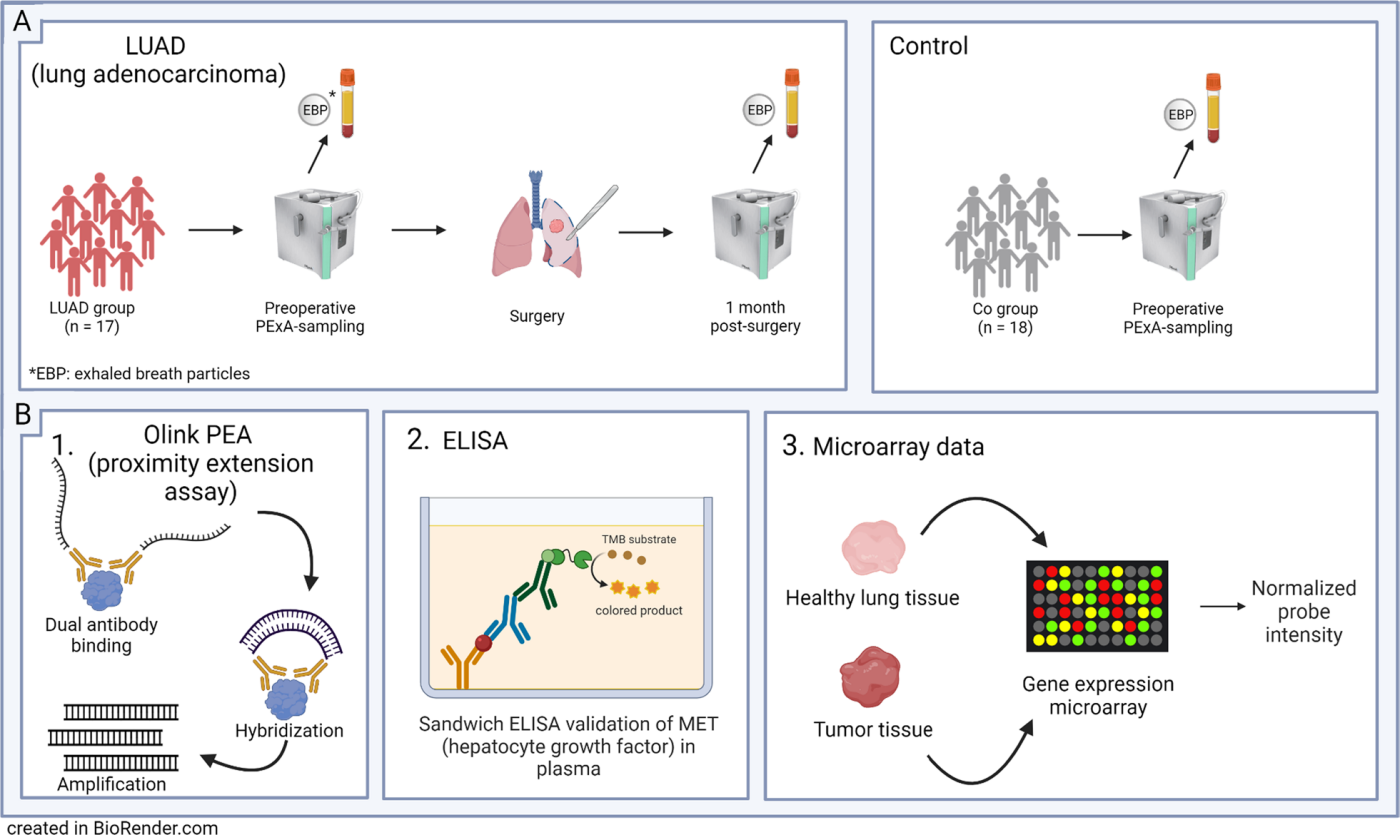
Overview of workflow. **A** Patient enrollment, describes sampling of exhaled breath particles (EBP) and plasma from lung adenocarcinoma patients (LUAD) and non-cancer surgical controls (Co). Samples were collected preoperatively for both cohorts and at 1-month post-surgery for the LUAD cohort. **B** Validation, (1) Proximity extension assay (PEA) technology. (2) Standard sandwich enzyme-linked immunosorbent assay with specific antibodies for hepatocyte growth factor (MET) in plasma from all patients and timepoints (n = 49). (3) Deposited microarray data were used for validation of proteins in EBP

**Table 2:** Staging, resection type and radicality

	n = 17
Histopathological classification	
Adenocarcinoma	17 (100)
Tumor stage	
IA	10 (59)
IB	4 (24)
IIA	1 (6)
IIB	0 (0)
IIIA	2 (12)
Lung resection	
Segmental resection	2 (12)
Lobectomy	15 (88)
R0	16 (94)
R1	1 (6)

Histopathological stage, type of resection and radicality in lung adenocarcinoma patients. All data reported as n (%). *R0* microscopic radicality, *R1* microscopic margins positive for tumor

### Collection of particles in exhaled air

The device to measure particles in exhaled air (PExA 2.0, PExA AB, Gothenburg, Sweden) contains an optical particle counter connected to an impactor for collection of EBP using a standardized breathing maneuver as described previously in detail [10]. Particles were quantified and divided into eight size bins ranging from 0.41 to 4.55 μm in diameter. Number of particles (count, n) and total accumulated mass (ng) were measured. PFR was described as particles per liter of exhaled air. Particles were collected onto a membrane (Millipores LCR membrane, Merck KGaA, Darmstadt, Germany) for biochemical analysis. In the LUAD group, sampling was carried out at two timepoints: the day before surgery (n = 15), and 1 month postoperatively (n = 16). Control patients were sampled at one timepoint, the day before surgery. An overview of the study is shown in Fig. 1.

### Analysis of exhaled breath particles

The Olink Target 96 Cardiometabolic panel (Olink Proteomics AB, Uppsala, Sweden) was used to analyze 92 proteins with the proximity extension assay (PEA), according to the manufacturer’s instructions [21]. Proteins with less than 15% detectability were excluded according to Olink’s predetermined limit of detection (LOD). The analysis is based on a calculated normalized protein expression (NPX), a relative protein quantification unit on a log_2_ scale. This allows for identification of changes in individual protein levels across the sample set. A high NPX value indicates a high protein concentration. NPX values are relative and thus cannot be compared between proteins. All EBP samples were analyzed at the same time.

### Heatmap

A heatmap was created in R version 4.1.3 using the packages “readxl” and gplots”. The data were standardized to allow comparison between the proteins and the protein levels were expressed as the z-score for each sample.

### Blood sampling

Blood was collected on the day before and 1 month after surgery in the LUAD group and the day before surgery in the control group. The blood was collected in ethylenediaminetetraacetic acid (EDTA) tubes (BD Vacutainer, Becton, Dickenson and company, Franklin Lakes, USA). The samples were centrifuged for 10 min at 5000 rpm, at 22 °C, within 30 min of collection, and the plasma was thereafter stored at −80 °C until analysis.

### MET in plasma using ELISA

An enzyme-linked immunosorbent assay (ELISA) targeting soluble MET (s-MET) (HGFR/c-MET ELISA kit (ELH-HGFR-1), RayBiotech Life Inc., Atlanta, GA, US) was performed on plasma according to the manufacturer’s instructions.

### Validation of proteins

The GSE10072 dataset which contains deposited microarray data describing gene expression in biopsies from primary LUAD (n = 58) and lung biopsies from healthy controls residing in the same area (n = 49) was selected from the Gene Expression Omnibus (GEO; https://www.ncbi.nlm.nih.gov/gds) to validate protein expression in EBP [22]. Unit of gene expression was expressed as normalized probe intensity (NPI).

### Statistical analysis

A power calculation with a statistical power of 87% and an effect size of 1.82 as calculated by hedges g was performed, using the results of biochemical analysis of surfactant A of a previously published study [10]. Descriptive statistics are presented as mean, range and standard error of mean (SEM). Student’s t-test, simple linear regression and Pearson’s correlation test were performed using GraphPad Prism version 9.2.0 for Windows (GraphPad Software, San Diego, California USA). Significance was defined as: p < 0.0001 (****), p < 0.001 (***), p < 0.01 (**), p < 0.05 (*), and p > 0.05 (not significant, ns).

## Results

### Particle flow rate from the airways was significantly higher in patients with LUAD

**Fig. 2:**
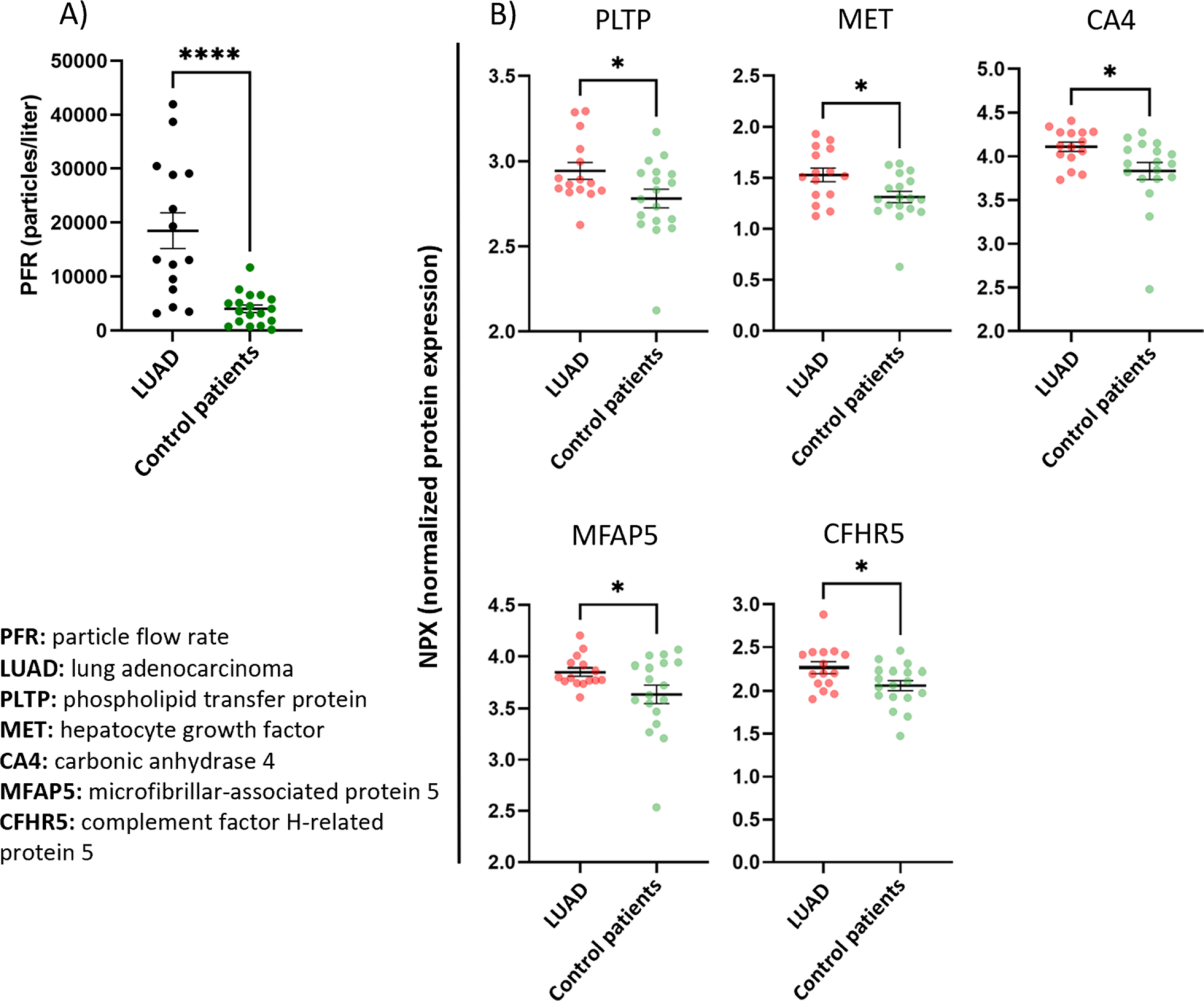
Particle flow rate (PFR) from the airways as well as five proteins in exhaled breath particles (EBP) were significantly higher in patients with lung adenocarcinoma (LUAD) compared to patients without LUAD. **A** PFR was significantly higher in patients with LUAD. **B** The Figure shows normalized protein expression (NPX) of phospholipid transfer protein (PLTP), hepatocyte growth factor (MET), carbonic anhydrase 4 (CA4), microfibrillar-associated protein 5 (MFAP5) and complement factor H-related protein 5 (CFHR5) before surgical removal of LUAD compared to patients without LUAD (control patients). Data are presented as mean ± SEM. Significance was defined as: p < 0.0001 (****), p < 0.001 (***), p < 0.01 (**), p < 0.05 (*), and p > 0.05 (not significant, ns)

Particle flow rate (PFR) was measured before and 1 month after surgery and was compared to patients without LUAD. A significantly higher PFR was seen among LUAD patients before surgery compared to the control patients (18,490 ± 3306 particles/L in LUAD patients, 4021 ± 899 particles/L in the control group [p < 0.0001]), (Fig. 2b). The PFR was still significantly increased 1 month after surgery compared to the patients without LUAD (p = 0.0001). Comparing PFR in LUAD patients before and after surgery, no significant difference was found (18,490 ± 3306 particles/L before surgery and 30,210 ± 6500 particles/L 1 month after surgery (p = 0.1142).

### Proteomic analysis of exhaled breath particles revealed differential levels between LUAD and patients without LUAD

**Fig. 3:**
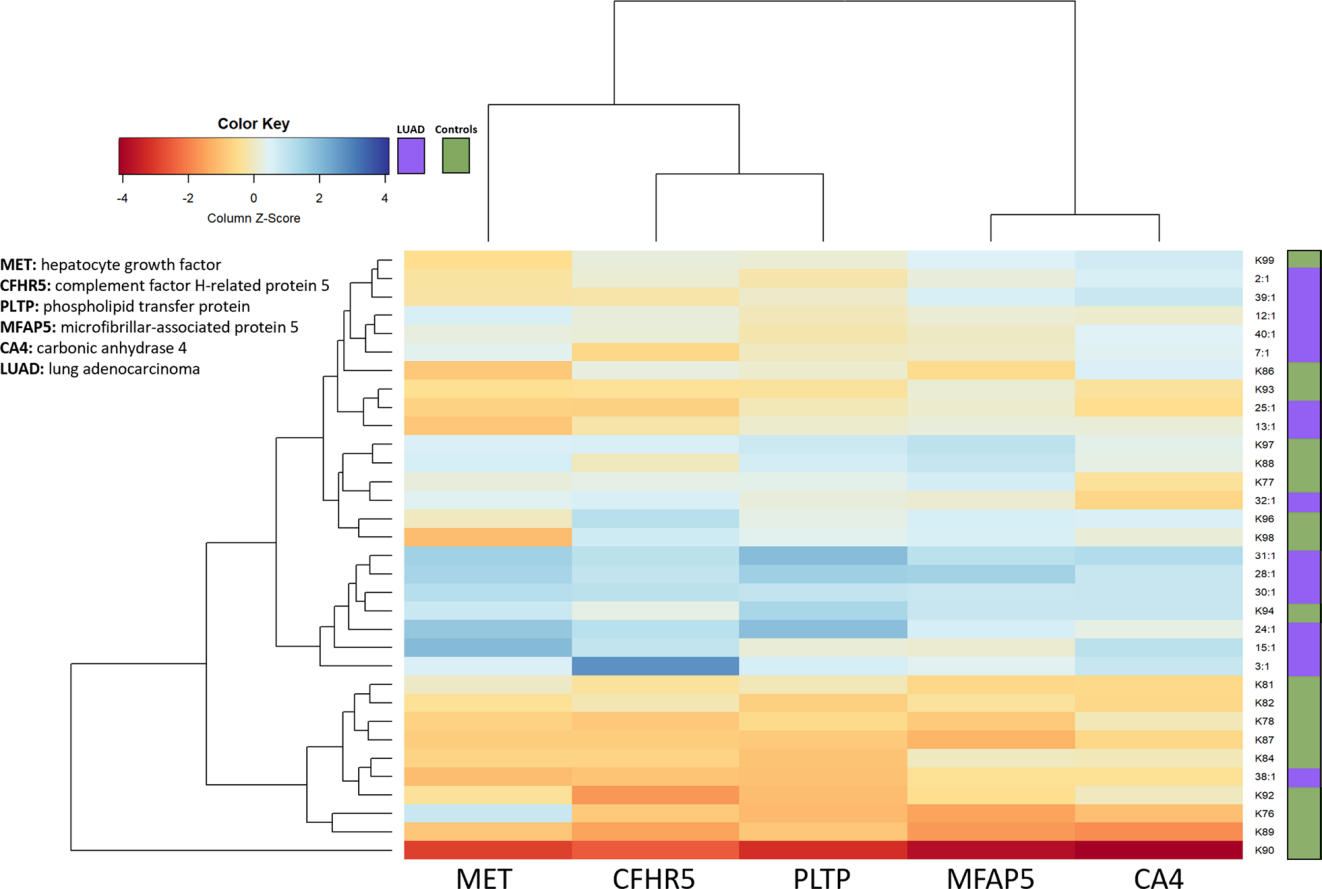
Clustering of five proteins in exhaled breath particles (EBP) from lung adenocarcinoma patients compared to control patients. The heatmap displays levels of hepatocyte growth factor receptor (MET), complement factor H-related protein 5 (CFHR5), phospholipid transfer protein (PLTP), microfibrillar-associated protein 5 (MFAP5) and carbonic anhydrase 4 (CA4) expressed as z-score on the x-axis. The y-axis displays the individual sample identification codes

A total of 89 unique proteins were detected using PEA technology and all were found in more than 75% of samples. A table of all 89 detected proteins can be found in Additional file 2: Table S1. Five proteins had a significantly higher protein concentration before surgery in the LUAD group compared to patients without LUAD: complement factor H-related protein 5 (CFHR5), microfibrillar-associated protein 5 (MFAP5), phospholipid transfer protein (PLTP), hepatocyte growth factor receptor/mesenchymal epithelial transition (HGF-R/MET) and carbonic anhydrase 4 (CA4), (Fig. 2a). These five proteins clustered as shown in the heatmap, the orange color representing a lower z-score (Fig. 3). One month after surgery, the three proteins, PLTP, MET and MFAP5, showed a decreasing trend, and when compared to patients without LUAD, the significance was no longer found, possibly indicating tumor removal.

### MET protein expression in plasma was elevated in LUAD patients

**Fig. 4:**
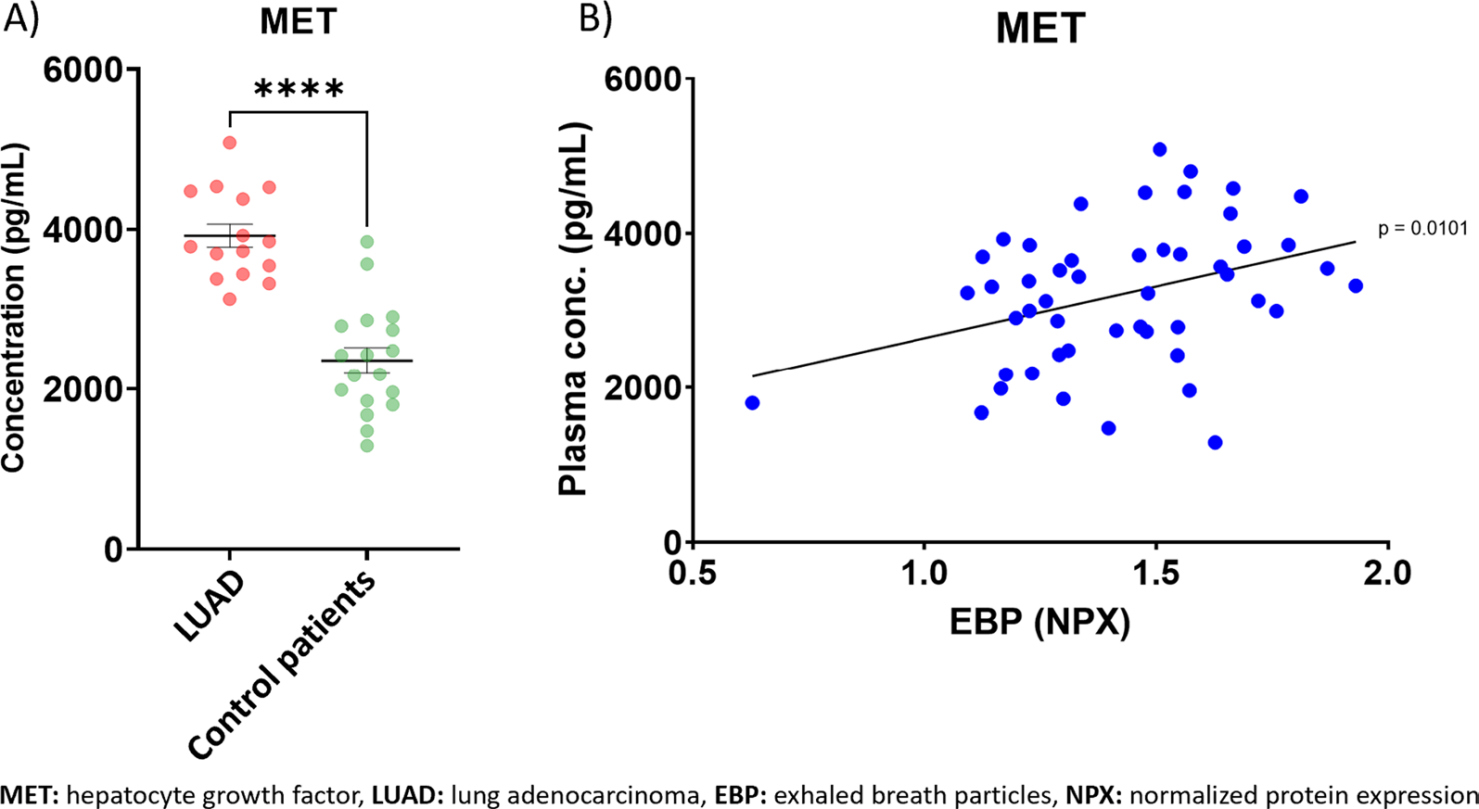
MET concentrations in plasma correlates to levels in exhaled breath particles (EBP). **A** Concentration of hepatocyte growth factor (MET) in plasma in patients with lung adenocarcinoma (LUAD) and patients without LUAD (control patients). Data are presented as mean ± SEM. **B** Correlation of MET in plasma and EBP for all patients at all timepoints (number of patients 35, number of samples 49) are shown. Protein level in EBP is expressed as normalized protein expression (NPX). Significance was defined as: p < 0.0001 (****), p < 0.001 (***), p < 0.01 (**), p < 0.05 (*), and p > 0.05 (not significant, ns)

To further confirm if our findings in EBP also could be detected in plasma, MET in plasma was analyzed using ELISA. A significantly higher MET concentration was found before surgery in the LUAD group compared to the control group (3921 ± 144 pg/mL in LUAD patients, 2358 ± 161 pg/mL in patients without LUAD [p < 0.0001]), in line with the findings in EBP (Fig. 4a). There was a significant correlation of protein levels in EBP and plasma in all patients (Fig. 4b).

### Survival

Postoperative 3-year survival follow-up was carried out for LUAD patients. All patients except one were still alive at 3 years.

### Validation of EBP data using dataset GSE10072 microarray data

Deposited microarray data from the dataset GSE10072 were used to validate the EBP findings. The proteins PLTP (gene ID NM_006227) and MET (gene ID BG170541 and BE870509) were significantly elevated in LUAD tissue compared to lung tissue from non-LUAD patients (PLTP: 9.66 ± 0.10 NPI in LUAD patients, 9.29 ± 0.12 NPI in non-LUAD patients [p = 0.008], MET BG170541: 9.52 ± 0.19 NPI in LUAD patients, 8.79 ± 0.07 NPI in non-LUAD patients [p = 0.0008], MET BE870509: 8.16 ± 0.07 NPI in LUAD patients, 7.88 ± 0.03 NPI in non-LUAD patients [p = 0.003]) (Table 3).

**Table 3:** Gene expression of the proteins PLTP and MET

Protein	GenBank	Gene expression cancer	Gene expression control	Significance
PLTP	NM_006227	9.66 ± 0.10	9.29 ± 0.12	p = 0.0080
MET	BG170541	9.52 ± 0.19	8.79 ± 0.07	p = 0.0008
MET	BE870509	8.16 ± 0.07	7.88 ± 0.03	p = 0.0030

Dataset GSE10072 from Gene Expression Omnibus compares tissue from patients with LUAD (n = 58) and healthy control patients (n = 49). Gene expression was calculated by microarray techniques and expressed as normalized probe intensity

## Discussion

This study explored the potential use of proteomics, based on EBP to detect possible biomarkers to diagnose patients with LUAD. In the present study patients undergoing lung cancer surgery were compared to patients without LUAD with similar age and smoking history. Using the PExA system, the PFR as well as proteins in EBP were analyzed in the two groups. The PFR reflects the number of exhaled particles and has the advantage of providing the examiner with an immediate result. The PFR was more than four times higher in the LUAD patients before the surgery compared to control patients indicating that PFR alone could be used to differentiate between patients with and without LUAD. Over the course of 1 month after surgery the PFR decreased among the LUAD patients, which might indicate tumor removal.

Given that protein content in EBP samples is considerably small, in the range of nanograms, the subsequent analysis of the EBP protein composition in the current study was performed using a unique technology, the proximity extension assay (PEA), which enables a robust high-throughput, multiplex immunoassay in very small biological samples. The technology is built upon pre-designed protein panels. Most of the PEA panels require some extent of dilution of the sample. In this early study, Olink’s cardiometabolic panel, which is the most sensitive panel and thus requires the least amount of biospecimen, was selected for technical reasons. The panel includes proteins involved in cellular metabolism, adhesion, and immunological processes. Patients with LUAD had significantly higher levels of PLTP, CA4, CFHR5, MFAP5, and MET compared to the control patients without LUAD, indicating that these proteins have a diagnostic potential for LUAD using EBP. One month after removal of the LUAD, expression of MET, PLTP and MFAP5 all decreased, which could be attributed to the radical tumor removal. All proteins that were found to be significantly higher expressed in EBP have a clear connection to cancer physiology, especially MET. More information on the involvement of the studied proteins is laid out in the Additional file 3. High levels of MET in plasma have been correlated with a significantly poorer overall survival [23]. A significant correlation was found between MET in EBP and s-MET in plasma among all LUAD patients before surgery. No correlation between mortality and levels of MET could be seen in the current study. One month after surgery, both EBP and plasma levels of MET decreased but were still higher than MET in EBP and plasma in patients without LUAD. Our results are in line with previous findings of MET in LUAD tumor tissue and plasma [24]. However, this is the first time MET has been detected in EBP. MET in EBP seems to reflect MET in blood in our cohort; however, MET in plasma does not have to be disease specific whilst MET in EBP specifically comes from the RTLF and thus processes in the lung and cannot be traced to other processes or malignancies in other organs in the body [25–28].

PLTP was among the other significant proteins found in EBP, which is expressed in different types of neoplasms and is involved in cancer development [29]. In the present study, significantly higher levels of PLTP were seen in LUAD patients compared to control patients before surgical removal of the lung cancer; however, after surgery the levels of PLTP had decreased and there was no longer any significant difference when compared to the control patients, potentially indicating successful removal of the tumor. CA4 was also found to be significantly different, and low expression of CA4 can promote proliferation of cancer cells, whereas overexpression can suppress proliferation of cancer cells [30]. Surprisingly, higher levels of CA4 were found in EBP samples in LUAD patients both preoperatively and after surgical removal of the tumor in the present study compared to control patients, indicating that CA4 might not be a good candidate for evaluating LUAD in EBP. In the current study significantly higher levels of MFAP5 were seen in EBP from LUAD patients before surgery compared to patients without LUAD; however, 1 month after surgery, the levels of MFAP5 had decreased, which might indicate that MFAP5 may be used as a biomarker in EBP to evaluate the successful surgical removal of LUAD. MFAP5 is known for signaling through the notch signaling pathway that activates cell proliferation and promotes the epithelial mesenchymal transition which leads to enhanced motility, invasion, and potential for metastasis in NSCLC [31–33]. Among the identified proteins PLTP and MET, as well as PFR were found significant, and based on the literature and support of external data these biomarkers were suggested as diagnostics in EBP for LUAD. However, some other proteins found are interesting but potentially less significant in EBP. CFHR5 is a protein in a family of 5, where CFHR1 previously been correlated to LUAD but not CFHR5. While defective CFHR5 may contribute to atypical hemolytic uremic syndrome, CFHR1 has been shown to be downregulated in tissue from LUAD tumors compared to healthy adjacent tissue. Given that CFHR1 was not included in the protein panel applied in this study, CFHR1 could not be analyzed. Given these unclarities, CFHR1 was not chosen for validation in the plasma. CA4 and MFAP5 showed reverse expression in EBP in the LUAD cohort then expected, in addition the two proteins could not be validated in any external data. Given these results CA4 and MFAP5 was seen as a less suitable biomarker in EBP.

To validate EBP findings we used microarray data from biopsies of healthy lung tissue and from LUAD tissue deposited in the dataset GSE10072. In this dataset, the gene expression of both PLTP and MET was higher in LUAD tissue compared to healthy lung tissue. These results are in line with the findings of the present study and support the use of those two biomarkers for diagnosing LUAD and to evaluate the successful surgical removal of LUAD. However, the levels of mRNA do not always correlate fully to corresponding proteins levels and tumors are very heterogeneous which indicate that further studies are of importance.

## Conclusion

For the first time, PFR was measured and EBP were collected and analyzed in LUAD patients with the potential to identify novel biomarkers for the diagnosis and prognosis of LUAD. The PFR alone enabled the possibility of distinguishing between LUAD and patients without LUAD. Collection of EBP made it possible to perform a proteomic analysis of the respiratory system. Here, the proteins PLTP and MET were significantly higher in LUAD patients, which was further validated with microarray data from LUAD biopsies in a separate cohort. PLTP and MET have been identified as potential biomarkers for LUAD diagnosis and in the evaluation of successful tumor excision.

## Strengths and limitations

The strength of this study is that it explores a novel technique of sampling proteins from particles in exhaled air. The technique itself has an advantage in that it samples particles exclusively from the small airways directly onto a membrane and thus dilution is avoided. Studies exploring exhaled breath proteomics in lung cancer are scarce and here we present both pre- and postoperative results. Furthermore, collection of EBPs is a completely non-invasive method, without risk to the patient. The non-invasiveness of the method makes it possible for non-clinicians to perform the sampling, which is advantageous in a screening setting. However, the current study does not explore the differences in PFR depending on the size of the tumor, nor how early one may detect lung cancer using this method. However, these questions will be further explored in future studies.

## Supplementary Information

**Additional file 1: Figure S1.** Flow char tof enrolled subjects. A total of 35 patients were enrolled in the study: 17 with lung adenocarcinoma (LUAD) and 18 non-cancer surgical controls. The LUAD group was sampled at two timepoints, once before surgery and once 1 month after surgery. The control group was sampled once before surgery. Every sampling includes collection of exhaled breath particles (EBP) and blood plasma. All collected plasma samples were analyzed with an ELISA to validate the expression of the protein hepatocyte growth factor (MET).

**Additional file 2: Table S1. **

**Additional file 3.** Discussion

## Data Availability

The dataset supporting the conclusions of this article is available in the Gene Expression Omnibus (GEO) repository, [https://www.ncbi.nlm.nih.gov/sites/GDSbrowser].

## Abbreviations

CA4: Carbonic anhydrase 4
CFHR5: Complement factor H-related protein 5
EBC: Exhaled breath condensate
EBP: Exhaled breath particles
EDTA: Ethylenediaminetetraacetic acid
HGF-R/MET: Hepatocyte growth factor receptor/mesenchymal epithelial transition
HGF: Hepatocyte growth factor
LUAD: Lung adenocarcinoma
MET: Hepatocyte growth factor receptor
MFAP5: Microfibrillar-associated protein 5
NPI: Normalized probe intensity
NSCLC: Non-small cell lung cancer
PEA: Proximity extension assay
PExA: Particles in exhaled air
PFR: Particle flow rate
PLTP: Phospholipid transfer protein
RTLF: Respiratory tract lining fluid
s-MET: Soluble MET

## References

[1] Sung H, Ferlay J, Siegel RL, Laversanne M, Soerjomataram I, Jemal A (2021). Global Cancer Statistics 2020: GLOBOCAN estimates of incidence and Mortality Worldwide for 36 cancers in 185 countries. CA Cancer J Clin.

[2] Gunnar Wagenius MH, Karin Olsson, Kristina Lamberg-Lundström, Stefan Bergström. Lungcancer Nationell Kvalitetesrapport för 2020. Nationella Lungcancerregistret, NLCR. 2020. https://doi.org/https://cancercentrum.se/globalassets/cancerdiagnoser/lunga-och-lungsack/kvalitetsregister/rapport/nlcr_nationell_rapport2020.pdf. Accessed 10 Nov 2022.

[3] de Koning HJ, van der Aalst CM, de Jong PA, Scholten ET, Nackaerts K, Heuvelmans MA (2020). Reduced lung-Cancer mortality with volume CT screening in a Randomized Trial. N Engl J Med.

[4] National Lung Screening Trial Research Team (2019). Lung cancer incidence and mortality with extended follow-up in the national lung screening trial. J Thorac Oncol.

[5] Mazzone PJ, Silvestri GA, Patel S, Kanne JP, Kinsinger LS, Wiener RS (2018). Screening for Lung Cancer: CHEST Guideline and Expert Panel Report. Chest.

[6] Oudkerk M, Devaraj A, Vliegenthart R, Henzler T, Prosch H, Heussel CP (2017). European position statement on lung cancer screening. Lancet Oncol.

[7] Toumazis I, Bastani M, Han SS, Plevritis SK (2020). Risk-based lung cancer screening: a systematic review. Lung Cancer.

[8] Salgia R (2017). MET in Lung Cancer: Biomarker Selection based on scientific rationale. Mol Cancer Ther.

[9] Drilon A, Cappuzzo F, Ou S-HI, Camidge DR (2017). Targeting MET in Lung Cancer: will expectations finally be MET?. J Thorac Oncol.

[10] Broberg E, Andreasson J, Fakhro M, Olin A-C, Wagner D, Hyllén S (2020). Mechanically ventilated patients exhibit decreased particle flow in exhaled breath as compared to normal breathing patients. ERJ Open Research.

[11] Amann A, Costello Bde L, Miekisch W, Schubert J, Buszewski B, Pleil J (2014). The human volatilome: volatile organic compounds (VOCs) in exhaled breath, skin emanations, urine, feces and saliva. J Breath Res.

[12] Patsiris S, Exarchos T, Vlamos P (2020). Exhaled Breath Condensate (EBC): is it a viable source of biomarkers for Lung Diseases?. Adv Exp Med Biol.

[13] Bajaj P, Ishmael FT (2013). Exhaled Breath Condensates as a source for biomarkers for characterization of Inflammatory Lung Diseases. J Anal Sci Methods Instrum.

[14] Broberg E, Hyllén S, Algotsson L, Wagner DE, Lindstedt S (2019). Particle flow profiles from the airways measured by PExA Differ in lung transplant recipients who develop primary graft dysfunction. Exp Clin Transplant.

[15] Stenlo M, Hyllén S, Silva IAN, Bölükbas DA, Pierre L, Hallgren O (2020). Increased particle flow rate from airways precedes clinical signs of ARDS in a porcine model of LPS-induced acute lung injury. Am J Physiol Lung Cell Mol Physiol.

[16] Hallgren F, Stenlo M, Niroomand A, Broberg E, Hyllén S, Malmsjö M (2021). Particle flow rate from the airways as fingerprint diagnostics in mechanical ventilation in the intensive care unit: a randomised controlled study. ERJ Open Res..

[17] Broberg E, Pierre L, Fakhro M, Algotsson L, Malmsjö M, Hyllén S (2019). Different particle flow patterns from the airways after recruitment manoeuvres using volume-controlled or pressure-controlled ventilation. Intensive Care Med Exp.

[18] Stenlo M, Silva IAN, Hyllén S, Bölükbas DA, Niroomand A, Grins E (2021). Monitoring lung injury with particle flow rate in LPS- and COVID-19-induced ARDS. Physiol Rep.

[19] Soares M, Mirgorodskaya E, Koca H, Viklund E, Richardson M, Gustafsson P (2018). Particles in exhaled air (PExA): non-invasive phenotyping of small airways disease in adult asthma. J Breath Res.

[20] Goldstraw P, Crowley J, Chansky K, Giroux DJ, Groome PA, Rami-Porta R (2007). The IASLC Lung Cancer Staging Project: proposals for the revision of the TNM stage groupings in the forthcoming (seventh) edition of the TNM classification of malignant tumours. J Thorac Oncol.

[21] Olink Cardiometabolic Validation Data. Olink proteomics AB. 2018. https://doi.org/https://www.olink.com/content/uploads/2021/09/olink-cardiometabolic-validation-data-v2.0.pdf. Accessed 10 Nov 2022.

[22] Landi MT, Dracheva T, Rotunno M, Figueroa JD, Liu H, Dasgupta A, et al. Cigarette smoking effect on lung adenocarcinoma. 2008. https://doi.org/https://www.ncbi.nlm.nih.gov/sites/GDSbrowser?acc=GDS3257#details. Accessed 10 Nov 2022.

[23] Gao HF, Li AN, Yang JJ, Chen ZH, Xie Z, Zhang XC (2017). Soluble c-Met levels correlated with tissue c-Met protein expression in patients with Advanced Non-Small-Cell Lung Cancer. Clin Lung Cancer.

[24] Lv H, Shan B, Tian Z, Li Y, Zhang Y, Wen S (2015). Soluble c-Met is a reliable and sensitive marker to detect c-Met expression level in lung cancer. Biomed Res Int.

[25] Bake B, Larsson P, Ljungkvist G, Ljungström E, Olin AC (2019). Exhaled particles and small airways. Respir Res..

[26] Lin J, Ma L, Zhang D, Gao J, Jin Y, Han Z (2019). Tumour biomarkers—tracing the molecular function and clinical implication. Cell Prolif.

[27] Mayeux R, Biomarkers (2004). Potential uses and limitations. NeuroRX.

[28] Almstrand AC, Josefson M, Bredberg A, Lausmaa J, Sjovall P, Larsson P (2012). TOF-SIMS analysis of exhaled particles from patients with asthma and healthy controls. Eur Respir J.

[29] Albers JJ, Vuletic S, Cheung MC (2012). Role of plasma phospholipid transfer protein in lipid and lipoprotein metabolism. Biochim Biophys Acta.

[30] Yu DH, Huang JY, Liu XP, Ruan XL, Chen C, Hu WD (2020). Effects of hub genes on the clinicopathological and prognostic features of lung adenocarcinoma. Oncol Lett.

[31] Yuan X, Wu H, Han N, Xu H, Chu Q, Yu S (2014). Notch signaling and EMT in non-small cell lung cancer: biological significance and therapeutic application. J Hematol Oncol.

[32] Sharif A, Shaji A, Chammaa M, Pawlik E, Fernandez-Valdivia R (2020). Notch transduction in non-small cell lung cancer. Int J Mol Sci..

[33] Database GTHG. MFAP5 Gene Protein Coding GeneCards The Human Gene Database. https://doi.org/https://www.genecards.org/cgi-bin/carddisp.pl?gene=MFAP5. Accessed 28 Jul 2021.

