# Supplementary material for: Exhaled phospholipid transfer protein and hepatocyte growth factor receptor in lung adenocarcinoma
Source: Respir Res. 2022 Dec 21;23:369. doi: 10.1186/s12931-022-02302-4 (PMC9768396; doi:10.1186/s12931-022-02302-4)
Supplement: Supplementary file 1 — Additional file 1: Figure S1. Flow char tof enrolled subjects. A total of 35 patients were enrolled in the study: 17 with lung adenocarcinoma (LUAD) and 18 non-cancer surgical controls. The LUAD group was sampled at two timepoints, once before surgery and once 1 month after surgery. The control group was sampled once before surgery. Every sampling includes collection of exhaled breath particles (EBP) and blood plasma. All collected plasma samples were analyzed with an ELISA to validate the expression of the protein hepatocyte growth factor (MET). [file 12931_2022_2302_MOESM1_ESM.zip › 12931_2022_2302_MOESM1_ESM/Additional figure 1 legend 221110.docx]

**Additional file 1: Figure S1**

**Flow chart of enrolled subjects.** A total of 35 patients were enrolled in the study: 17 with lung adenocarcinoma (LUAD) and 18 non-cancer surgical controls. The LUAD group was sampled at two timepoints, once before surgery and once 1 month after surgery. The control group was sampled once before surgery. Every sampling includes collection of exhaled breath particles (EBP) and blood plasma. All collected plasma samples were analyzed with an ELISA to validate the expression of the protein hepatocyte growth factor (MET).
