# Supplementary material for: Exhaled phospholipid transfer protein and hepatocyte growth factor receptor in lung adenocarcinoma
Source: Respir Res. 2022 Dec 21;23:369. doi: 10.1186/s12931-022-02302-4 (PMC9768396; doi:10.1186/s12931-022-02302-4)
Supplement: Supplementary file 2 — Additional file 2: Table S1. [file 12931_2022_2302_MOESM2_ESM.docx]

**Additional file 2: Table S1**

|  | **Protein** | **UniProt ID** | **Protein name** |
| --- | --- | --- | --- |
| 1. | PRCP | P42785 | Lysosomal Pro-X carboxypeptidase |
| 2. | CA1 | P00915 | Carbonic anhydrase 1 |
| 3. | ICAM1 | P05362 | Intercellular adhesion molecule 1 |
| 4. | CHL1 | O00533 | Neural cell adhesion molecule L1-like protein |
| 5. | TGFBI | Q15582 | Transforming growth factor-beta-induced protein ig-h3 |
| 6. | ENG | P17813 | Endoglin |
| 7. | PLTP | P55058 | Phospholipid transfer protein |
| 8. | SERPINA7 | P05543 | Thyroxine-binding globulin |
| 9. | IGFBP3 | P17936 | Insulin-like growth factor-binding protein 3 |
| 10. | CR2 | P20023 | Complement receptor type 2 |
| 11. | SERPINA5 | P05154 | Plasma serine protease inhibitor |
| 12. | FCGR3B | O75015 | Low affinity immunoglobulin gamma Fc region receptor III-B |
| 13. | IGFBP6 | P24592 | Insulin-like growth factor-binding protein 6 |
| 14. | CDH1 | P12830 | Cadherin-1 |
| 15. | CCL5 | P13501 | C-C motif chemokine 5 |
| 16. | CCL14 | Q16627 | C-C motif chemokine 14 |
| 17. | GNLY | P22749 | Granulysin |
| 18. | NOTCH1 | P46531 | Neurogenic locus notch homolog protein 1 |
| 19. | PAM | P19021 | Peptidyl-glycine alpha-amidating monooxygenase |
| 20. | PROC | P04070 | Vitamin K-dependent protein C |
| 21. | CST3 | P01034 | Cystatin-C |
| 22. | NCAM1 | P13591 | Neural cell adhesion molecule 1 |
| 23. | PCOLCE | Q15113 | Procollagen C-endopeptidase enhancer 1 |
| 24. | LILRB1 | Q8NHL6 | Leukocyte immunoglobulin-like receptor subfamily B member 1 |
| 25. | MET | P08581 | Hepatocyte growth factor receptor |
| 26. | LTBP2 | Q14767 | Latent-transforming growth factor beta-binding protein 2 |
| 27. | IL7R | P16871 | Interleukin-7 receptor subunit alpha |
| 28. | VCAM1 | P19320 | Vascular cell adhesion protein 1 |
| 29. | SELL | P14151 | L-selectin |
| 30. | F11 | P03951 | Coagulation factor XI |
| 31. | COMP | P49747 | Cartilage oligometric matrix protein |
| 32. | CA4 | P22748 | Carbonic anhydrase 4 |
| 33. | PTPRS | Q13332 | Receptor-type tyrosine-protein phosphatase S |
| 34. | MBL2 | P11226 | Mannose-binding protein C |
| 35. | TIMP1 | P01033 | Metalloproteinase inhibitor 1 |
| 36. | ANGPTL3 | Q9Y5C1 | Angiopoietin-related protein 3 |
| 37. | REG3A | Q06141 | Regenerating islet-derived protein 3-alpha |
| 38. | SOD1 | P00441 | Superoxide dismutase [Cu-Zn] |
| 39. | CD46 | P15529 | Membrane cofactor protein |
| 40. | ITGAM | P11215 | Integrin alpha-M |
| 41. | TNC | P24821 | Tenascin |
| 42. | NID1 | P14543 | Nidogen-1 |
| 43. | CFHR5 | Q9BXR6 | Complement factor H-related protein 5 |
| 44. | SPARCL1 | Q14515 | SPARC-like protein 1 |
| 45. | PLXNB2 | O15031 | Plexin-B2 |
| 46. | MEGF9 | Q9H1U4 | Multiple epidermal growth factor-like domains protein 9 |
| 47. | ANG | P03950 | Angiogenin |
| 48. | ST6GAL1 | P15907 | Beta-galactoside alpha-2,6-sialyltransferase 1 |
| 49. | DPP4 | P27487 | Dipeptidyl peptidase 4 |
| 50. | FCN2 | Q15485 | Ficolin-2 |
| 51. | FETUB | Q9UGM5 | Fetuin-B |
| 52. | CES1 | P23141 | Liver carboxylesterase 1 |
| 53. | CRTAC1 | Q9NQ79 | Cartilage acidic protein 1 |
| 54. | TCN2 | P20062 | Transcobalamin-2 |
| 55. | PRSS2 | P07478 | Trypsin-2 |
| 56. | ICAM3 | P32942 | Intercellular adhesion molecule 3 |
| 57. | SAA4 | P35542 | Serum amyloid A-4 protein |
| 58. | CNDP1 | Q96KN2 | Beta-Ala-His dipeptidase |
| 59. | FCGR2A | P12318 | Low affinity immunoglobulin gamma Fc region receptor II-a |
| 60. | NRP1 | O14786 | Neuropilin-1 |
| 61. | EFEMP1 | Q12805 | EGF-containing fibulin-like extracellular matrix protein 1 |
| 62. | TIMD4 | Q96H15 | T-cell immunoglobulin and mucin domain-containing protein 4 |
| 63. | FAP | Q12884 | Prolyl endopeptidase FAP |
| 64. | TIE1 | P35590 | Tyrosine-protein kinase receptor Tie-1 |
| 65. | THBS4 | P35443 | Thrombospondin-4 |
| 66. | F7 | P08709 | Coagulation factor VII |
| 67. | GP1BA | P07359 | Platelet glycoprotein Ib alpha chain |
| 68. | LYVE1 | Q9Y5Y7 | Lymphatic vessel endothelial hyaluronic acid receptor 1 |
| 69. | CA3 | P07451 | Carbonic anhydrase 3 |
| 70. | TGFBR3 | Q03167 | Transforming growth factor beta receptor type 3 |
| 71. | DEFA1 | P59655 | Neutrophil defensin 1 |
| 72. | CD59 | P13987 | CD59 glycoprotein |
| 73. | APOM | O95445 | Apolipoprotein M |
| 74. | OSMR | Q99650 | Oncostatin-M-specific receptor subunit beta |
| 75. | LILRB2 | Q8N423 | Leukocyte immunoglobulin-like receptor subfamily B member 2 |
| 76. | UMOD | P07911 | Uromodulin |
| 77. | CCL18 | P55774 | C-C motif chemokine 18 |
| 78. | COL18A1 | P39060 | Collagen alpha-1(XVIII) chain |
| 79. | LCN2 | P80188 | Neutrophil gelatinase-associated lipocalin |
| 80. | KIT | P10721 | Mast/stem cell growth factor receptor Kit |
| 81. | C1QTNF1 | Q9BXJ1 | Complement C1q tumor necrosis factor-related protein 1 |
| 82. | GAS6 | Q14393 | Growth arrest-specific protein 6 |
| 83. | IGLC2 | P0DOY2 | Ig lambda-2 chain C regions |
| 84. | PLA2G7 | Q13093 | Platelet-activating factor acetylhydrolase |
| 85. | TNXB | P22105 | Tenascin-X |
| 86. | MFAP5 | Q13361 | Microfibrillar-associated protein 5 |
| 87. | VASN | Q6EMK4 | Vasorin |
| 88. | LILRB5 | O75023 | Leukocyte immunoglobulin-like receptor subfamily B member 5 |
| 89. | C2 | P06681 | Complement C2 |

Proteins detected in EBP (n = 89) using Olink proteomics Cardiometabolic protein panel.
