# Supplementary material for: Exhaled phospholipid transfer protein and hepatocyte growth factor receptor in lung adenocarcinoma
Source: Respir Res. 2022 Dec 21;23:369. doi: 10.1186/s12931-022-02302-4 (PMC9768396; doi:10.1186/s12931-022-02302-4)
Supplement: Supplementary file 3 — Additional file 3. Discussion [file 12931_2022_2302_MOESM3_ESM.docx]

**Exhaled phospholipid transfer protein and hepatocyte growth factor receptor in lung adenocarcinoma**

Jesper Andreasson, MD^1,2^, Embla Bodén, MD^1,2^, Mohammed Fakhro, MD, PhD^3^, Camilla von Wachter^4^, Franziska Olm, PhD^1,2^ Malin Malmsjö, MD, PhD^2^, Oskar Hallgren, PhD^2^, Sandra Lindstedt, MD, PhD^1, 2^

^1^Department of Cardiothoracic Surgery, Skåne University Hospital, Sweden

^2^Lund University, Department of Clinical Sciences, Sweden

^3^Department of Cardiothoracic Surgery, Rigshospitalet, University of Copenhagen, Denmark

^4^Ludwig-Maximilians-University, Munich, Germany

Corresponding author:

Sandra Lindstedt

Entrégatan 7, 22242, Lund, Sweden

[sandra.lindstedt_ingemansson@med.lu.se](mailto:sandra.lindstedt_ingemansson@med.lu.se)

**Additional file 3: Discussion**

The proteins studied in this paper are all linked to processes central to cancer such as inflammation, cell proliferation, metastasis or anti-tumor immune mediated responses. The over- or under expression of these protein biomarkers impacts upon cancer development and progression in different ways. A literature search was conducted to gain understanding of how each protein functions within this field.

PLTP (phospholipid transfer protein) is a glycoprotein that regulates transportation of phospholipids, and is highly expressed in pulmonary epithelial cells. PLTP is expressed in different types of neoplasms and is involved in cancer development (1). Silencing of PLTP in a murine model has been shown to result in increased inflammation, suggesting an anti-inflammatory role for PLTP. Expression of PLTP is elevated in patients with chronic obstructive pulmonary disease (COPD) and the activity of the protein correlates negatively with lung function as determined by forced expiratory volume in the first second (FEV1) (2).

CA4 (carbonic anhydrase 4) is a zinc membrane-associated metalloenzyme which catalyzes the interconversion of water and carbon dioxide into ions of carbonic acid and was also found in exhaled breath particles (EBP). CA4 is normally found in healthy lung tissue and has a role in regulating the local pH of the lung, but it is expressed in lower levels in lung cancer patients in general and in those with lung adenocarcinoma (LUAD) specifically (3-5). Lower levels have also been linked to lymph node metastasis and a shorter overall survival in lung cancer (5).

MFAP5 (microfibrillar-associated protein 5) is an extracellular microfibril-associated glycoprotein, upregulated in cancer-associated fibroblasts in multiple malignancies, including non-small cell lung cancer (NSCLC) (6, 7). Among others, MFAP5 is central to cancer-associated fibroblast differentiation and epithelial mesenchymal transition (7, 8). MFAP5 signals through the Notch signaling pathway that activates cell proliferation and promotes the epithelial mesenchymal transition which leads to enhanced motility, invasion, and potential for metastasis in NSCLC (8-10).

CFHR (complement factor H-related protein 5) is a family of five proteins, CFHR1–5, which can each bind to the complement component C3b. While defective CFHR5 may contribute to atypical hemolytic uremic syndrome, CFHR1 has been shown to be downregulated in tissue from LUAD tumors compared to healthy adjacent tissue. Patients with lower expressed levels of CFHR1 were found to have a significantly shorter overall survival (11).

MET (hepatocyte growth factor receptor) is a transmembrane receptor tyrosine kinase for hepatocyte growth factor (HGF) involved in epithelial cellular migration, proliferation, morphogenesis, and survival. Mutation of MET is linked to several forms of cancer, including NSCLC (12). Studies have shown that patients with elevated expression of MET intratumorally have significantly lower survival compared to patients with MET-negative tumors (13). Furthermore, MET amplification is involved in the acquired resistance that develops in many patients treated with epidermal growth factor receptor (EGFR) inhibitor (14).

**References**

1. Albers JJ, Vuletic S, Cheung MC. Role of plasma phospholipid transfer protein in lipid and lipoprotein metabolism. Biochim Biophys Acta. 2012;1821(3):345-57.

2. Brehm A, Geraghty P, Campos M, Garcia-Arcos I, Dabo AJ, Gaffney A, et al. Cathepsin G degradation of phospholipid transfer protein (PLTP) augments pulmonary inflammation. Faseb j. 2014;28(5):2318-31.

3. Xu Y, Xu WH, Shi SN, Yang XL, Ren YR, Zhuang XY, et al. Carbonic Anhydrase 4 serves as a Clinicopathological Biomarker for Outcomes and Immune Infiltration in Renal Cell Carcinoma, Lower Grade Glioma, Lung Adenocarcinoma and Uveal Melanoma. J Cancer. 2020;11(20):6101-13.

4. Yu DH, Huang JY, Liu XP, Ruan XL, Chen C, Hu WD, et al. Effects of hub genes on the clinicopathological and prognostic features of lung adenocarcinoma. Oncol Lett. 2020;19(2):1203-14.

5. Chen J, Hu L, Zhang F, Wang J, Chen J, Wang Y. Downregulation of carbonic anhydrase IV contributes to promotion of cell proliferation and is associated with poor prognosis in non-small cell lung cancer. Oncol Lett. 2017;14(4):5046-50.

6. Yeung TL, Leung CS, Yip KP, Sheng J, Vien L, Bover LC, et al. Anticancer Immunotherapy by MFAP5 Blockade Inhibits Fibrosis and Enhances Chemosensitivity in Ovarian and Pancreatic Cancer. Clin Cancer Res. 2019;25(21):6417-28.

7. Navab R, Strumpf D, Bandarchi B, Zhu CQ, Pintilie M, Ramnarine VR, et al. Prognostic gene-expression signature of carcinoma-associated fibroblasts in non-small cell lung cancer. Proc Natl Acad Sci U S A. 2011;108(17):7160-5.

8. Yuan X, Wu H, Han N, Xu H, Chu Q, Yu S, et al. Notch signaling and EMT in non-small cell lung cancer: biological significance and therapeutic application. J Hematol Oncol. 2014;7:87.

9. Sharif A, Shaji A, Chammaa M, Pawlik E, Fernandez-Valdivia R. Notch Transduction in Non-Small Cell Lung Cancer. Int J Mol Sci. 2020;21(16).

10. Database GTHG. MFAP5 Gene Protein Coding GeneCards The Human Gene Database [cited 2021 28/7]. Available from: <https://www.genecards.org/cgi-bin/carddisp.pl?gene=MFAP5>.

11. Wu G, Yan Y, Wang X, Ren X, Chen X, Zeng S, et al. CFHR1 is a potentially downregulated gene in lung adenocarcinoma. Mol Med Rep. 2019;20(4):3642-8.

12. Matsumoto K, Umitsu M, De Silva DM, Roy A, Bottaro DP. Hepatocyte growth factor/MET in cancer progression and biomarker discovery. Cancer Sci. 2017;108(3):296-307.

13. Masuya D, Huang C, Liu D, Nakashima T, Kameyama K, Haba R, et al. The tumour-stromal interaction between intratumoral c-Met and stromal hepatocyte growth factor associated with tumour growth and prognosis in non-small-cell lung cancer patients. Br J Cancer. 2004;90(8):1555-62.

14. Bean J, Brennan C, Shih JY, Riely G, Viale A, Wang L, et al. MET amplification occurs with or without T790M mutations in EGFR mutant lung tumors with acquired resistance to gefitinib or erlotinib. Proc Natl Acad Sci U S A. 2007;104(52):20932-7.
